# Antibody response to a new member of the DBL family (EBP2) after a brief *Plasmodium vivax* exposure

**DOI:** 10.1371/journal.pntd.0010493

**Published:** 2022-06-17

**Authors:** Bárbara A. S. Lima, Gabriela M. Fernandes, Letícia M. Torres, Camilla V. Pires, Jéssica R. S. Alves, Sâmick L. Moreira-Nascimento, Maria Fernanda A. Nascimento, Sofia L. Afonso, Helena L. Costa, Isabela P. Cerávolo, Tais N. Sousa, Irene S. Soares, Francis B. Ntumngia, John H. Adams, Luzia H. Carvalho, Flora S. Kano

**Affiliations:** 1 Biologia Molecular e Imunologia da Malária, Instituto René Rachou, Fundação Oswaldo Cruz, Belo Horizonte, Minas Gerais, Brazil; 2 Center for Global Health and Infectious Diseases Research, College of Public Health, University of South Florida, Tampa, Florida, United States of America; 3 Laboratório de Imunopatologia, Instituto René Rachou, Fundação Oswaldo Cruz, Belo Horizonte, Minas Gerais, Brazil; 4 Departamento de Análises Clínicas e Toxicológicas, Faculdade de Ciências Farmacêuticas, Universidade de São Paulo, São Paulo, Brazil; George Washington University School of Medicine and Health Sciences, UNITED STATES

## Abstract

*Plasmodium vivax* blood-stage invasion into reticulocyte is critical for parasite development. Thus, validation of novel parasite invasion ligands is essential for malaria vaccine development. Recently, we demonstrated that EBP2, a Duffy binding protein (DBP) paralog, is antigenically distinct from DBP and could not be functionally inhibited by anti-DBP antibodies. Here, we took advantage of a small outbreak of *P*.*vivax* malaria, located in a non-malarious area of Brazil, to investigate for the first time IgM/IgG antibodies against EBP2 and DEKnull-2 (an engineering DBPII vaccine) among individuals who had their first and brief exposure to *P*.*vivax* (16 cases and 22 non-cases). Our experimental approach included 4 cross sectional surveys at 3-month interval (12-month follow-up). The results demonstrated that while a brief initial *P*.*vivax* infection was not efficient to induce IgM/ IgG antibodies to either EBP2 or DEKnull-2, IgG antibodies against DEKnull-2 (but not EBP2) were boosted by recurrent blood-stage infections following treatment. Of interest, in most recurrent *P*. *vivax* infections (4 out of 6 patients) DEKnull-2 IgG antibodies were sustained for 6 to 12 months. Polymorphisms in the *ebp2* gene does not seem to explain EBP2 low immunogenicity as the *ebp2* allele associated with the *P*.*vivax* outbreak presented high identity to the original EBP2 isolate used as recombinant protein. Although EBP2 antibodies were barely detectable after a primary episode of *P*.*vivax* infection, EBP2 was highly recognized by serum IgG from long-term malaria-exposed Amazonians (range from 35 to 92% according to previous malaria episodes). Taken together, the results showed that individuals with a single and brief exposure to *P*.*vivax* infection develop very low anti-EBP2 antibodies, which tend to increase after long-term malaria exposure. Finally, the findings highlighted the potential of DEKnull-2 as a vaccine candidate, as in non-immune individuals anti-DEKnull-2 IgG antibodies were boosted even after a brief exposure to *P*.*vivax* blood stages.

## Author summary

Vaccines might be a crucial component of the current efforts to malaria control and elimination, and much of the vaccine-related research on *P*. *vivax* has been focused on region II of the Duffy binding protein (DBPII), a ligand for human blood-stage infection. Recently, the newly described Erythrocyte binding protein 2 (EBP2), a *P*.*vivax* DBP paralog that it is antigenically distinct from DBP, was identified as potential vaccine targets. To date, scarce data are available about the naturally acquired immunity to EBP2. In a small outbreak of *P*.*vivax* malaria, located in a non-malarious area, we investigated whether a first *P*.*vivax* exposure induces antibodies against EBP2 that could be boosted by *P*.*vivax* recurrent infections. In parallel, we included an engineered DBPII vaccine (named DEKnull-2) whose antibody response were previously associated with broadly neutralizing *P*.*vivax* antibodies. This study shows EBP2, compared with DEKnull-2, was poorly immunogenic among individuals who experienced their first blood-stage *P*. *vivax* malaria infection. However, EBP2 was highly immunogenic in long-term malaria exposed individuals, reinforcing its potential as a *P*. *vivax* blood-stage vaccine candidate. Finally, our results reinforce that multiple blood-stage antigens should be targeted for the development of efficient vaccines against *P*. *vivax*.

## Introduction

Malaria remains a major public health concern despite all efforts for control. The World Health Organization registered 241 million malaria cases in 2020 with an estimated 12% increase in death rate compared to 2019 [1]. Among the *Plasmodium* parasite that infect humans, *Plasmodium vivax* is the most widespread outside the African continent [1]. Increasing reports of severe disease caused by *P*. *vivax*, drug resistance, and recurrent relapses by reactivation of liver stage hypnozoites are causes for concern [2–7]. Therefore, developing an effective vaccine is needed for current efforts to malaria control and elimination.

The invasion of the erythrocytes by *Plasmodium* spp merozoite is a multistep process mediated by molecular interaction between erythrocyte receptors and parasite ligands, and it is essential for parasite development [8–10]. Thus, a vaccine capable of inducing neutralizing antibodies against these invasion ligands is critical to prevent parasite invasion and, consequently, human disease [11–14]. For *P*. *vivax*, the leading blood-stage vaccine candidate, the Duffy binding protein (region II, DBPII), is involved in the interaction between the parasite and its receptor on reticulocytes, the Duffy antigen/ receptor for chemokines (DARC) [15–17]. Although antibodies that can inhibit the DBPII-DARC interaction (BIAbs) are elicited only after a long-term exposure to malaria [18–22], high levels of BIAbs are associated with clinical protection [19,23]. Recently, a *P*. *vivax* DBP paralog, and novel member of Erythrocyte binding-like family, termed Erythrocyte binding protein 2 (EBP2), was identified in field isolates [24]. EBP2 shares the key domain features of other invasion ligands, including the region II or Duffy binding-like (DBL) [24] but it is antigenically distinct from DBP and could not be functionally inhibited by anti-DBP antibodies [25]. Of interest, we demonstrated EBP2 host cell specificity is more restricted than DBP binding and that EBP2 binds preferentially to Duffy-positive young (CD71^high^) reticulocytes [25], which suggest that EBP2 may be involved in an alternative *P*.*vivax* invasion pathway.

A recent study investigated the potential existence of synergistic or additive effects of combinations of antibody responses to a panel of 38 *P*. *vivax* antigens on the reduced risk of vivax malaria in children from Papua New Guinea (PNG) [26]. The results revealed that high level of antibodies against multiple antigens were associated with strong potential protective effect (PPE). In addition, the strongest PPE (> 90%) was observed using the combination among five antigens, including DBPII and EBP2 [26]. More interestingly, the additive effect of the antibodies against DBPII on the reduction clinical *P*. *vivax* malaria in children from PNG was associated with the antibodies against EBP2 [27], however, this association DBPII/ EBP2 was predominantly against a single DBPII allele from PNG. In fact, the region II of DBP (DBPII), which contains residues for receptor-binding, is highly polymorphic [28–30], with induction of antibody response that are biased to specific allele [31,32]. To overcome the inherent bias towards induction of strain-specific immune responses, an engineered DBPII vaccine, termed DEKnull-2, elicited a strong broadly neutralizing antibody response, including induction of long-term persistent antibody responses of naturally acquired immunity [33].

Scarce data are available on naturally acquired immunity to the newly described EBP2, most of which are restricted to Southeast Asian [26,27,34]. Considering the potential of the association between EBP2 and to the next generation of engineered DBPII immunogen (DEKnull-2) as vaccine against *P*. *vivax* blood-stage, we investigated here whether a first *P*. *vivax* exposure is able to induces antibodies against EBP2 and DEKnull-2, and if these responses could be boosted by *P*. *vivax* relapses/recurrence. This study took advantage of an outbreak of *P*. *vivax* malaria, in a non-endemic area in Brazil. We demonstrated that EBP2 was poorly immunogenic among individuals who experienced their first blood-stage *P*. *vivax* malaria infection compared with DEKnull-2. However, EBP2 was shown to be highly immunogenic in long-term malaria exposed individuals.

## Material and methods

### Ethics statement

The ethical and methodological aspects of this study were approved by the Ethical Committee of Research on Human Beings of the Institute René Rachou / FIOCRUZ Minas (No. 007/ 2006, No. 07/2009, No.12/2010, No. 26/2013, and CAAE 50522115.7.0000.5091). The study participants were informed about the aims and procedures of the study and voluntary participation solicited and agreed with voluntary participation through written formal consent. For the child participants, the written formal consent was obtained from the parent/guardian. The current study was conducted according to Laboratory biosafety and biosecurity policy guidelines of the Oswaldo Cruz Foundation (FIOCRUZ, Ministry of Health, Brazil (http://www.fiocruz.br/biosseguranca/Bis/manuais/biosseg_manuais.html).

### Study site and population

#### *P*. *vivax* malaria outbreak, participants, and sample collection

**Fig 1:**
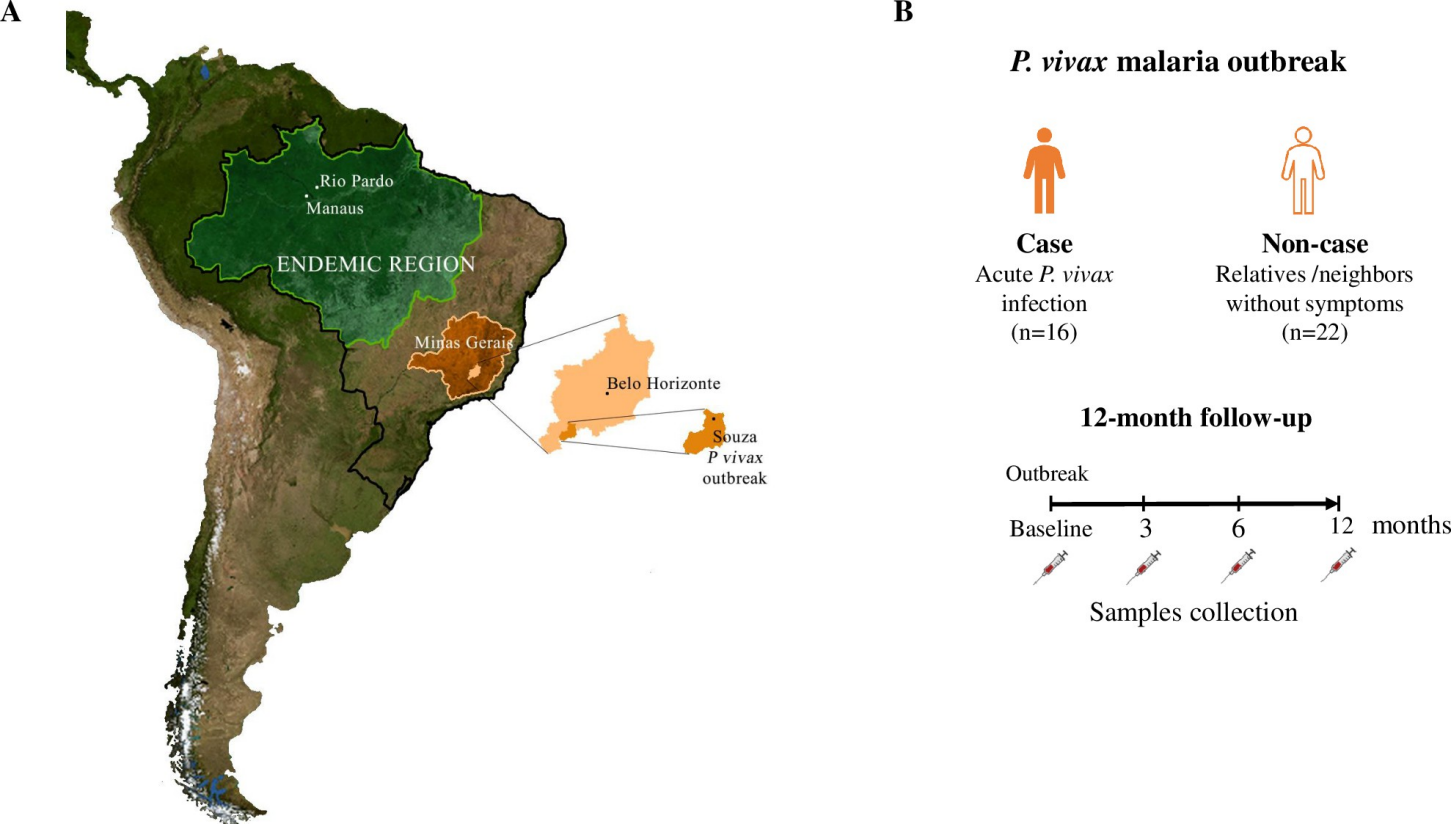
Localization of study site and design. (A) The Brazil map in South America showing the localization of the *P*. *vivax* malaria outbreak that occurred in small village named Souza, Minas Gerais state (orange), outside of the Amazonian endemic region for malaria in Brazil (grey), and 2000 km away from endemic region (green). (B) Study design of first and brief *P*. *vivax* exposure that included individuals with acute malaria infection confirmed by microscopy (Cases, n = 16), and relatives and/ or neighbors without malaria symptoms and negative microscopy diagnosis (non-Case, n = 22). The 12-month follow-up were initiated briefly after the beginning of outbreak (BL, baseline) with three additional and identical cross-sectional surveys carried-out at 3, 6 and 12 months after the first survey. Adapted from U.S. Geological Survey (USGS) (https://www.usgs.gov/).

This study was carried out in a small community, named Souza, that has about 1,100 inhabitants located on the banks of one of the arms of the Brumadinho dam, about 70 km far from Belo Horizonte, Minas Gerais State, a non-malarious endemic region of Brazil [31], Malaria has never been reported in this area before which is about 2,000 km away from the Brazilian malaria endemic region—Amazon region (Fig 1A). The source of the infection was a man from the community who returned infected by *P*. *vivax* from the Amazon region in January 2003, and experienced recurrence in the following months. The first human malaria case detected in the outbreak area, named S14, remained at the hospital for about 10 days, until a malaria diagnosis could be established. Because malaria infection had never been reported in the outbreak area previously, the physicians failed to consider malaria on presentation of this patient. The *P*. *vivax* malaria outbreak lasted for approximately 60 days (between April and May) in 2003, with the last malaria case diagnosed on 21 May 2003; since then, local/regional of Minas Gerais Departments of Health had maintained entomological and epidemiological surveillance of the area until the end of 2003. The entomological surveys incriminated the vector *Anopheles darling* as responsible for local malaria transmission [35]. Control activities also included an active search for acute malaria by thick blood smears and outdoor/ indoor spraying of residual insecticide (cypermethrine) [36]. During the outbreak, 25 individuals presented febrile symptoms such as fever, headache, myalgia and confirmed *P*. *vivax* infection by microscopy (Case). In that time, all patients were promptly treated with chloroquine (1.5 g for 3 days) plus primaquine (30 mg daily for 7 days) and followed-up. In the case of relapses and/ or recrudescence, a second round of treatment was administered (3-day course of chloroquine and a 15-day course of primaquine). Sixteen out of 25 cases (median 27 years, interquartile range, IRQ: 20–42) were enrolled in the current study, and 6 out of the 16 (38%) cases experienced one or two recurrent *P*. *vivax* infections. All *P*. *vivax* recurrent infection were confirmed by thick blood smears and DNA sequencing of a single *dbpII* allele [31]. In addition, 22 relatives and/or neighbors (median 24 years, IRQ: 20–45) who were exposed at the risk of *P*. *vivax* transmission but did not develop blood-stage infection were included as non-Cases (Fig 1B). The aims of the project were discussed with the community and after the written agreement consent 5 ml of whole blood were collected in EDTA vacutainer tube to obtain plasma and DNA from all participants. The followed-up were initiated briefly after the beginning of outbreak (Baseline), with three others cross-sectional surveys were carried-out 3, 6 and 12 months after the first survey.

#### Long-term malaria-exposed individuals

To evaluate the influence of time on malaria exposure and acquisition of naturally antibody response to recombinant *P*. *vivax* proteins, additional sera samples of individuals with long-term malaria exposure (median 17 years, IQR: 11–33) from a rural community of the Amazon rain forest (Rio Pardo, Amazonas) were included in the current study (Fig 1A). The details of the study site and malaria transmission patterns were described previously [22,37]. For the current study, we selected sera samples from individuals who had single or multiple clinical *P*. *vivax* malaria episodes, whose *P*. *vivax* infections were confirmed by microscopy (official malaria data available at the Brazilian Malaria Epidemiological Surveillance Information System–SIVEP- Malaria).

#### Recombinant blood-stage *P*. *vivax* antigens

*EBP2*. Recombinant EBP2, which includes amino acids 159–485 from C127 Cambodian isolate [24], was codon-optimized for expression in *Escherichia coli*, cloned into pET21a vector, with a C-terminal 6xHis tag. After expression, recombinant EBP2 was purified from inclusion bodies by affinity chromatography using Ni+ Sepharose 6 fast flow (GE Lifesciences), and refolded by a rapid dilution, resulting in a 37 kDa protein as previously described [25].

*DBPII-based antigens*. Recombinant engineered vaccine DEKnull-2 [33], based on domain II of *P*. *vivax* Duffy binding protein (DBPII) (243aa–573aa), and DBPII Souza isolate from the outbreak (DBPII-outbreak) [31] were expressed in *E*. *coli* as 39 kDa protein fusion with 6xHis tag and purified as previously described [33,38].

*MSP1-19*. The 19-kDa C-terminal fragment of the Merozoite Surface Protein-1 of *P*. *vivax* (MSP1-19), which represents amino acids 1616–1704 of the full-length MSP-1 polypeptide, was expressed as a 6xHis tag fusion protein and purified as described previously [39].

#### Serological assay

Plasma IgM and IgG antibodies level were measured by conventional ELISA [40,41]. Briefly, the concentration of all recombinant *P*. *vivax* proteins was previously titrated, and defined as 1.5μg/ml for EBP2, 3μg/ml for DBPII-outbreak and DEKnull-2, and 1μg/ml for MSP1-19. Plasma samples were diluted at 1:400 and 1:100 for IgM and IgG, respectively. Peroxidase-conjugated IgM and anti-IgG were used as secondary antibody at 1:5000 dilution. Results were expressed as ELISA reactivity index (RI) for each protein, calculated as the ratio of the mean optical density (OD at 492nm) of sample to the mean OD plus three standard deviations of 20–30 unexposed volunteers. Values of RI > 1.0 were considered positive.

#### Amplification and sequencing of *epb2* alleles from the outbreak

The primer sets used for the amplification and sequencing of *ebp2* gene of *P*. *vivax* from the outbreak isolate were designed using Cambodian field isolate (C127) as a reference (accession number: KC987954) [24]. To cover a larger DBL region of *ebp2* (979 bp) and to obtain high quality of the full *ebp2* sequence, we designed three sets of overlapping primers that covers a region beyond the DBL of EBP2, corresponding to nucleotides 201 to 1618 (aa 68–535) (S1 Fig). The PCR was performed using high fidelity platinum Taq DNA polymerase (Invitrogen Corporation, Carlsbad, CA) with the following primer sets: fragment 1 (F1: 5’-AGAAATAAGAAAAAGAGCAGTAG-3’) and (R1: 5’-ATTTCCATGCGCCACGATG-3’); fragment 2 (F2: 5’-CAAGTCCTTCTTTCACTAAAC-3’), and (R2:5’-GTATCCCATTGCTCCTTCTTTA-3’) and fragment 3 (F3: 5’-AGGTAAAGGCAAAGAAGGCA-3’) and (R3: 5’-CTCTTCCTTTACTCTTCCCA-3’). The PCR products were purified using the ExoSAP-IT PCR Product Cleanup (Affymetrix, USB) and sequenced by Sanger method directly using BigDye Terminator v3.1 Cycle Sequencing Kit (Applied Biosystems—Life Technologies) and ABI 3730xL DNA analyzer (Applied Biosystems). The sequences were analyzed using Bioedit Sequence Alignment Editor (http://www.mbio.ncsu.edu/BioEdit/bioedit.html) and Chromas version 2.6.6 (http://technelysium.com.au/wp/chromas/). A total of six reads of each fragment was used for alignment and construction of the sequence contig of *ebp2* (EBP2 outbreak) which was compared to the reference sequence of C127 isolate.

## Statistical analysis

The graphics and analysis were performed using GraphPad Prism version 9 (www.graphpad.com) and the R statistical software (version 3.3.2). Differences in proportions were evaluated by chi-square (χ^2^) or Fisher’s exact tests, as appropriate. Shapiro-Wilk test was performed to evaluate the normality distribution of variables. Differences in means or medians of antibody levels among the groups were performed using one-way ANOVA or Kruskal-Wallis test followed by Tukey’s or Dunn’s post hoc test, as appropriate. Multivariate logistic regression models were built to describe independent associations between covariates (age, gender, number of previous malaria episodes, and recent malaria) and antibodies against EBP2 and DEKnull-2. All analyses were considered statistically significant at the 5% level (P < 0.05).

## Results

### IgM and IgG antibody responses to DBL- antigens after the first *P*. *vivax* exposure

In the *P*. *vivax* outbreak study area, we evaluated the IgM and IgG antibodies against two members of the *P*. *vivax* DBL family, the novel EBP2 and the engineered DBPII based vaccine (DEKnull-2). In addition, the well-characterized *P*. *vivax* MSP1-19 was included as a highly immunogenic *P*. *vivax* blood-stage antigen. The demographic, epidemiological and immunological data at enrollment of case (*P*. *vivax* infection, n = 16) and non-case (negative relative and/ or neighbors, n = 22) are summarized in Table 1. At the baseline, one out of 16 (6%) cases showed detectable IgM antibodies response to EBP2 and DEKnull-2 while nine out of 16 cases (56%) were positive for IgM antibody response to MSP1-19. Non-cases had barely IgM response to DEKnull-2 and MSP1-19. For IgG antibody response, 69% (11 out of 16) of the cases showed IgG response to MSP1-19, followed by 6% to DEKnull-2. There were no detectable IgG antibodies against EBP2. As expected, no one of non-cases showed detectable IgG response.

**Table 1:** Demographic, epidemiological, and immunological data of individuals in the *P*. *vivax* malaria outbreak area at enrollment.

Characteristics		Case^1^ (n = 16)	Non-Case^2^ (n = 22)
Age, years (median, IQR^3^)		27 (20–42)	24 (20–45)
Gender, male: female ratio		2:1	0.4:1
Antibody response^4^, n (%)			
IgM	EBP2	1 (6%)	0 (0%)
	DEKnull-2	1 (6%)	1 (5%)
	MSP1-19	9 (56%)	1 (5%)
IgG	EBP2	0 (0%)	0 (0%)
	DEKnull-2	1 (6%)	0 (0%)
	MSP1-19^5^	11 (69%)	0 (0%)

^1^ confirmed symptomatic *P*. *vivax* infection (positive blood-smears by microscopy diagnosis)

^2^ relatives and/or neighbors exposed at the risk of *P*. *vivax* transmission during the outbreak without symptoms or blood parasites

^3^ IQR = Interquartile Range

^4^ evaluated by conventional ELISA using recombinant *P*. *vivax* proteins

^5^ according to [31]

**Fig 2:**
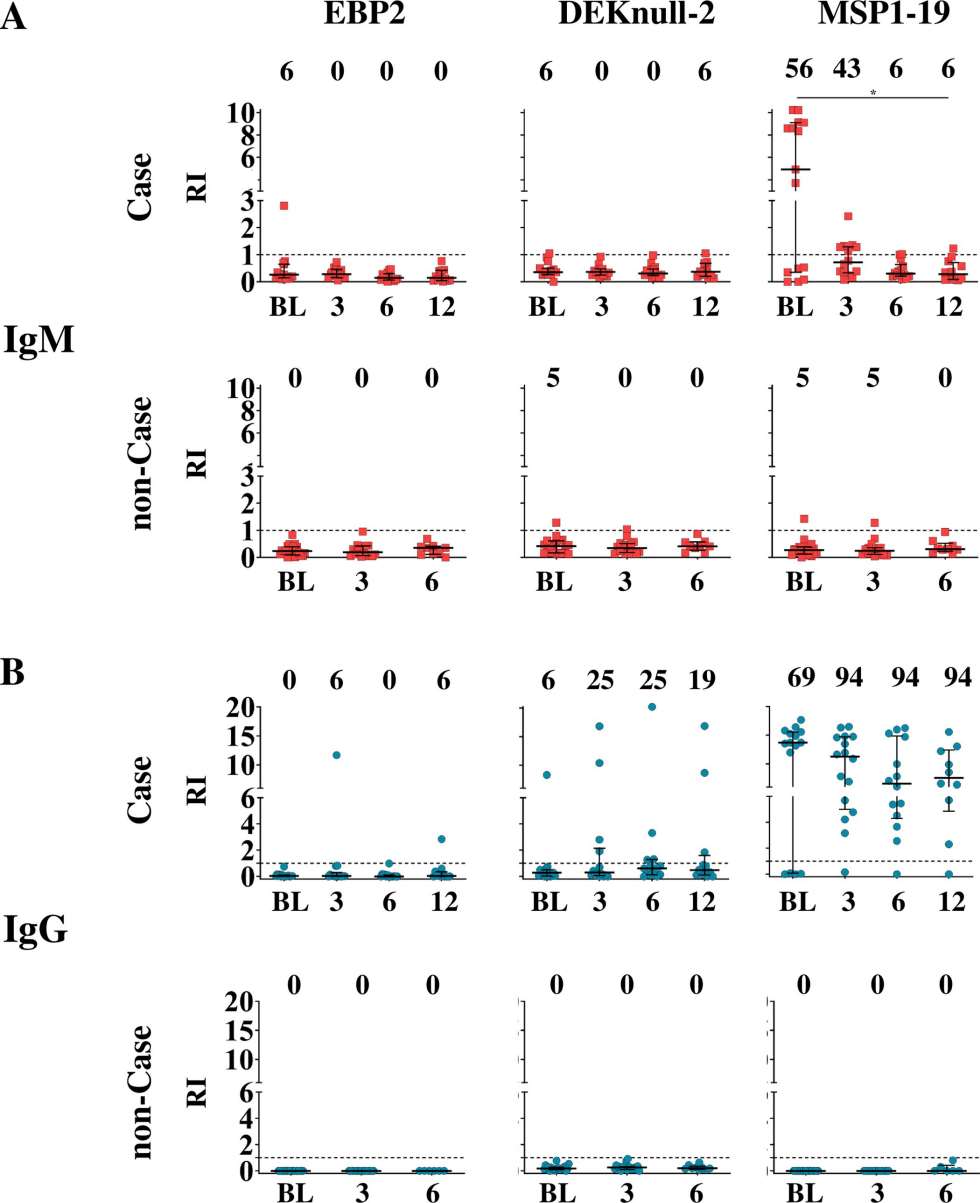
IgM and IgG antibodies responses to *P*. *vivax* antigens after the first *P*. *vivax* exposure. (A) Frequency and level of IgM antibodies against *P*. *vivax* EBP2, DEKnull-2 and MSP1-19 in individuals with acute *P*. *vivax* infection (Case, n = 16), and relatives and/ or neighbors without malaria symptoms (non-Case, n = 22). (B) Frequency and level of IgG antibody against *P*. *vivax* EBP2, DEKnull-2 and MSP1-19 in Case and non-Case. The IgM and IgG antibody responses were evaluated at the outbreak (BL, baseline), 3, 6 and 12 months after the *P*. *vivax* outbreak for Case group, and at BL, 3, 6-month for non-Case group. Sera reactivity was expressed as ELISA Reactivity Index (RI). Percentage (%) of antigen-specific IgM and IgG positive was expressed at the top of the graph. The dashed line represents RI = 1. Samples with RI > 1.0 are considered positive. Differences statistically significant were indicate by asterisk (*P<0.05, ** P<0.01, *** P<0.001, **** P<0.0001).

Further, the IgM and IgG antibodies against all antigens studied were evaluated over time, including 3, 6, and 12-month after the enrollment (Fig 2). Barely detectable IgM antibody responses to either EBP2 or DEKnull-2 were observed over the 12-month follow-up period (Fig 2A). A similar result was observed with the DBPII allele associated with the *P*. *vivax* outbreak (S2A Fig). In *P*. *vivax* cases, while IgM antibody response to MSP1-19 was 56% at the baseline, frequencies and levels of responders sharply decreased after the 3^rd^ month of the follow-up (43% to 6% from 3 to 12 months, respectively; Chi-square, p<0.001) (Fig 2A, right top panel). In non-case, IgM antibody response to all antigens were not detected, except a barely response to MSP1-19 (Fig 2A, bottom panel).

For IgG antibodies against EBP2, a single *P*. *vivax* case (one out of 16, 6%) showed detectable antibodies at 3 and 12-month of follow-up (Fig 2B, first left panel). The proportion of DEKnull-2 IgG responders was much higher at 3-month of follow-up (4 out 16, 25%), and remained stable over the follow-up period. Considering the DBPII-outbreak allele, the proportion of DBPII responders was similar to that of DEKnull-2 at 3-month, however, the level of DBPII IgG antibodies was lower and the proportion of DBPII IgG responders fluctuated over 12 months (S2A Fig). As expected, the proportion of MSP1-19 IgG responders was more than 90% with high IgG levels (Fig 2B, right panel). In non-cases, no detectable *P*. *vivax*-specific IgG antibodies was observed at any time of the observational period (Fig 2B, bottom panel).

### *The ebp2* allele amplification and sequencing

**Fig 3:**
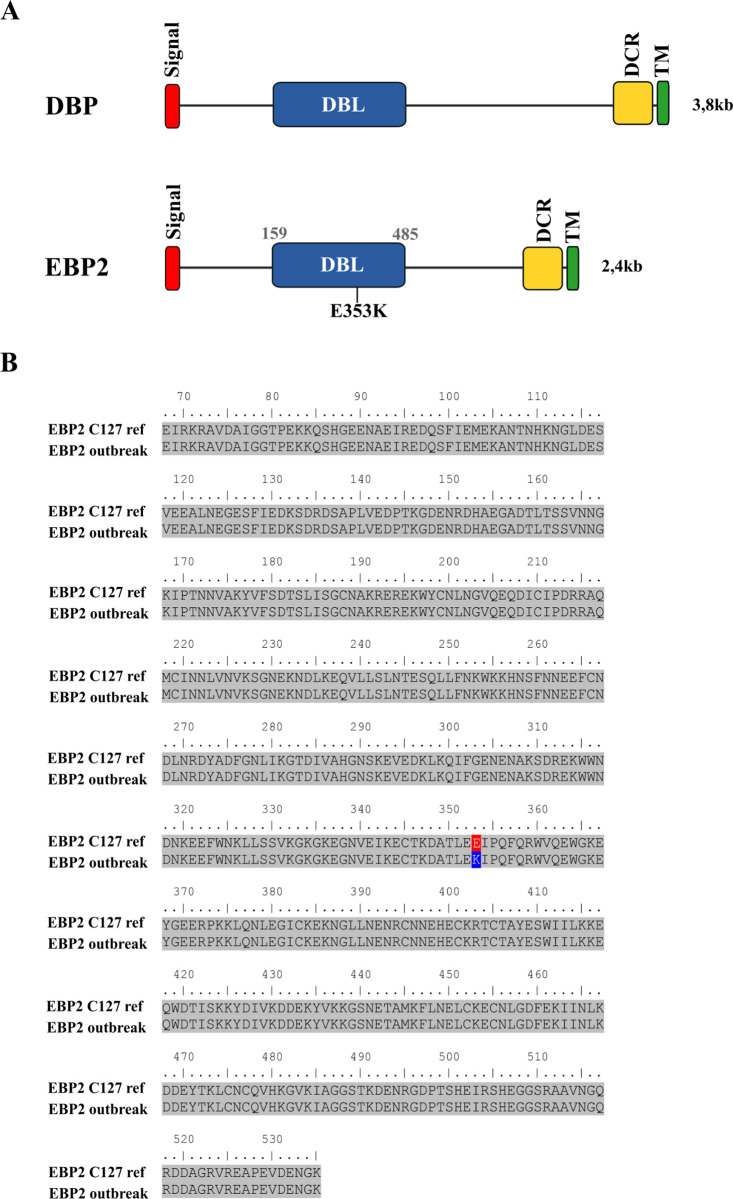
Schematic representation of *ebp2* structure and amino acid sequence alignment of *P*. *vivax* outbreak isolate. (A) Schematic representation of DBPII and EBP2 domains. The signal peptide (red box), Duffy-binding-like domain (DBL) (blue box), C-terminal cysteine-rich domain (DCR) (yellow box) and transmembrane domain (TM) (green box) are indicated. The polymorphic site in EBP2 is indicated in DBL region (E353K). (B) Amino acid sequence alignment of the DBL domain of *P*. *vivax* EBP2 of outbreak isolate (S17 sample). The sequence found at position 353 is highlighted.

Previously, DNA sequences from primary/recurrent *P*. *vivax* outbreak samples identified a single *dbpII* allele among samples from the outbreak area [31]. Here, we sequenced the full DBL domain of EBP2 (region II) that shares common features characteristics with members of DBL family (Fig 3A). A sequence alignment of the full amino acid sequences of the EBP2 DBL domain (aa 159 to 485) with the reference sequence of the Cambodian *P*. *vivax* C127 isolate [24], showed a very high sequence similarity between isolates with a single amino acid substitution of Glu-Lys at position 353 (E353K), resulting from a non-synonymous nucleotide polymorphism at position 1057 (G1057A), (Fig 3B).

### Influence of *P*. *vivax* infection recurrence on IgM and IgG antibody responses to DBL-antigens

**Fig 4:**
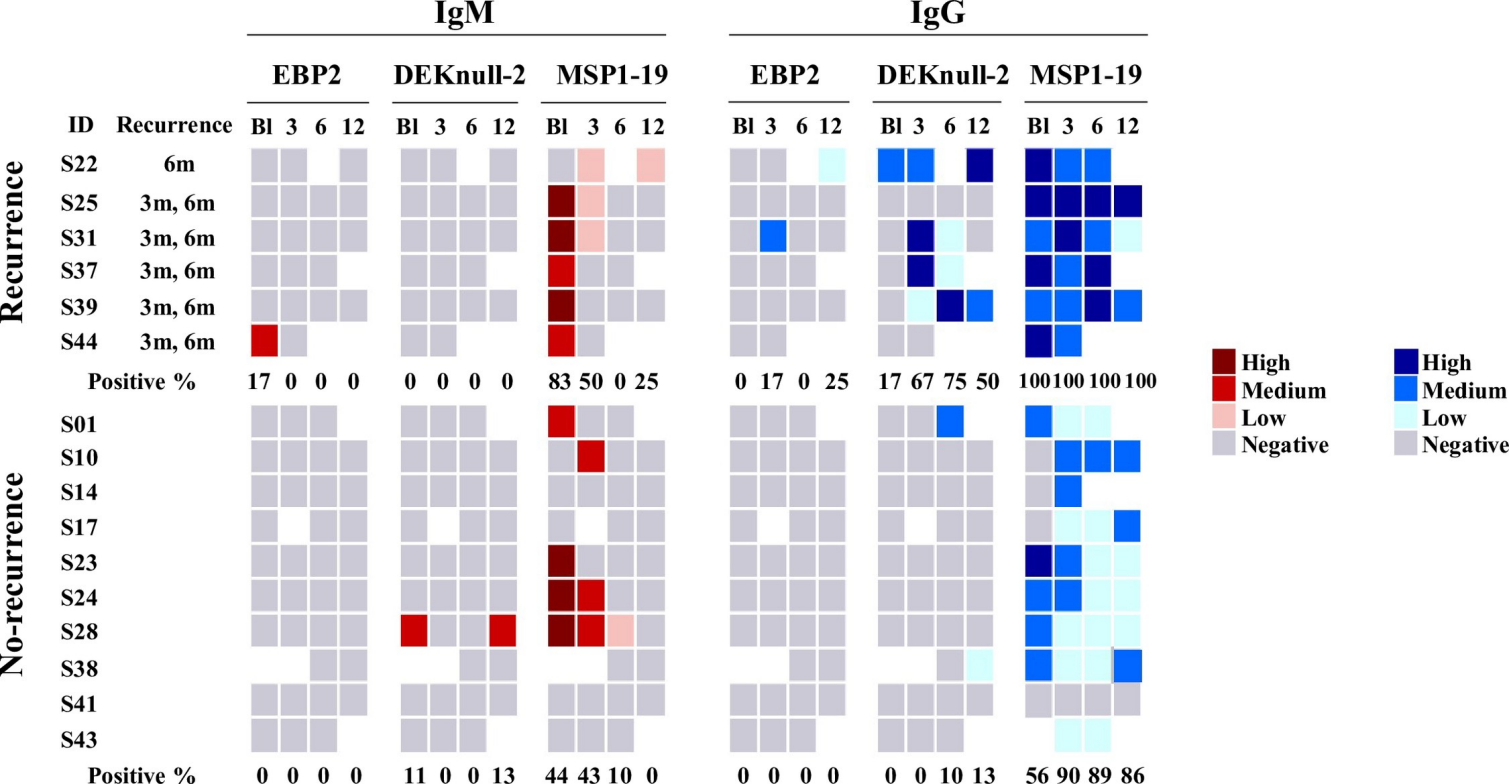
Influence of recurrence on the IgM and IgG responses to distinct *P*.*vivax* vaccine candidates. Individuals who experienced first *P*. *vivax* malaria infection (Cases) were grouped into: (i) Recurrence (n = 6)–individuals who experienced one or two additional recurrent *P*. *vivax* infection; and (ii) No-recurrence (n = 10)–individuals who did not have additional blood-stage *P*. *vivax* infection. IgM (red) and IgG (blue) antibodies responses to EBP2, DEKnull-2 and MSP1-19 were measured by ELISA at baseline (BL), 3, 6, and 12-month after the outbreak. The color gradient indicates the intensity of IgM (red) and IgG (blue) antibody levels categorized by tercile in High (Upper tercile), Medium (Second tercile) and Low (First tercile) for each protein and parasitemia (orange) according to parasitemia range. The time points of follow-up study and recurrent *P*. *vivax* infection moment were indicated at the heatmap.

Next, we sought to investigate the influence of *P*. *vivax* recurrent infections on naturally acquired antibody response to EBP2, DEKnull-2 and MSP1-19 (Fig 4). Individuals who experienced their primary blood-stage *P*. *vivax* infection were divided into two subgroups: (i) *Recurrence*–individuals who experienced at least one additional episode of blood-stage *P*. *vivax* infection after their primary clinical attack (n = 6); (ii) *No-recurrence*–cases who did not have additional *P*. *vivax* blood-stage infections (n = 10).

In *P*. *vivax* recurrent infections, it was not possible to detect booster effect on EBP2 or DEKnull-2 IgM antibodies, as all individuals remained with undetectable antibody response (Fig 4). A different profile was obtained for MSP1-19, in which most individuals responded with increased titers of IgM antibodies. Considering IgG response, DEKnull-2 (but not EBP2) showed a significant booster response during the recurrence (Fig 4, right panel); more specifically, most recurrent *P*. *vivax* cases were associate with increased levels of anti-DEKnull-2 IgG antibodies, resulting in the proportion of positive ranging from 50 to 75%. However, no-Recurrence subgroup had low frequency of DEKnull-2 IgG responders (0 to 13%). The profile of IgG antibodies to the DBPII-outbreak allele was similar to DEKnull-2, although the frequency and intensity of IgG response to DBPII seems to decrease more rapidly (S2B Fig). In recurrence subgroup, parasitemia did not associated with booster response to both DEKnull-2 or DBPII (S3 Fig).

Overall, it was not possible to detect booster effect on EBP2 or DEKnull-2 IgM antibodies, but recurrent *P*. *vivax* infections influenced IgG antibody response to DEKnull-2.

### Risk factors associated with IgG antibody response to EBP2 and DEKnull-2

The evaluation of naturally acquired antibody response to EBP2 among individuals who experienced their first and brief *P*. *vivax* exposure suggested that EBP2 is a poorly immunogenic antigen. Thus, we hypothesized that *P*. *vivax* EBP2 specific IgG antibody response could be dependent on the long-term exposure to malaria. For that, we included additional sera from individuals with long-term exposure to unstable malaria transmission in the Amazon region, which were grouped as having a single or multiple clinical *P*. *vivax* episodes (microscopically confirmed).

**Fig 5:**
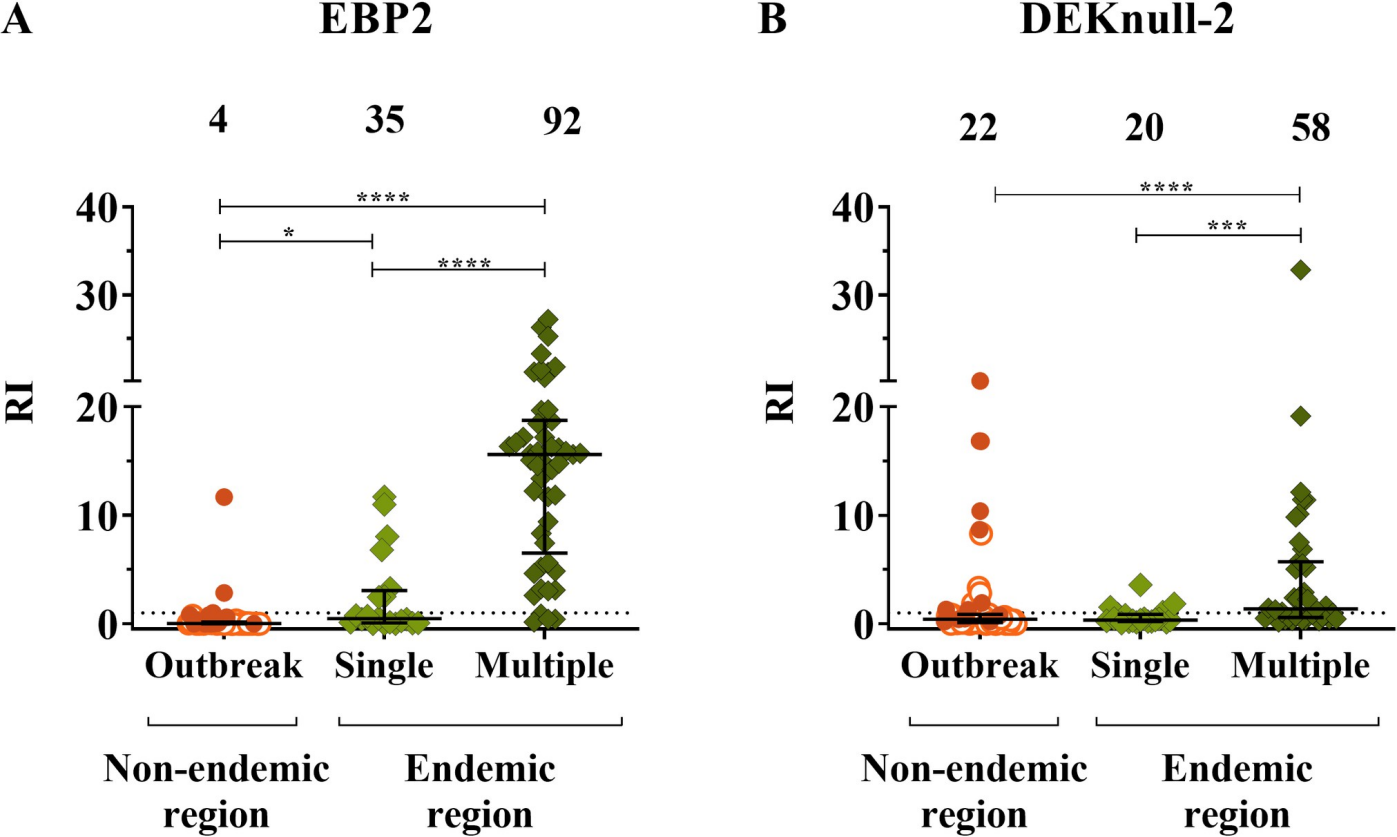
IgG response to distinct *P*.*vivax* DBL vaccine candidates among individuals with different previous malaria episodes. Frequency and levels of IgG antibody against *P*. *vivax* (A) EBP2 and (B) DEKnull-2 among (1) Outbreak (n = 55)—individuals from non-endemic region, including recurrent *P*. *vivax* infection (closed circle) and without recurrence (open circle) and, individuals long-term malaria exposure from endemic region with (2) Single previous malaria episode (n = 20) and multiple episodes (n = 49). Sera reactivity was expressed as ELISA Reactivity Index (RI). Samples with RI > 1.0 were considered positive. The percentage (%) of positive IgG antibody response to each protein was expressed at the top of the graph. Differences statistically significant were indicate by asterisk (*P<0.05, ** P<0.01, *** P<0.001, **** P<0.0001).

As compared with *P*. *vivax* malaria outbreak group, the proportion of EBP2 responders was quite different in individuals living in the malaria endemic region (Fig 5). The frequency of EBP2 IgG responders ranged from 35% (7/20) to 92% (45/ 49) between individuals who had had a single or multiple *P*. *vivax* malaria episodes (Chi-square, p<0.0001). Moreover, multiple malaria episodes were associated with much higher levels of IgG antibodies (p<0.0001). A similar profile of antibody response was observed with DEKnull-2 (Fig 5B).

Finally, we performed a multiple linear regression analysis adjusted by age, gender, number of previous malaria episode and time since the last malaria episodes, to evaluate which variables were associated with the risk to develop antibody response to each proteins studied (S1 Table). The number of previous malaria episodes increased by two-fold the chance of having a positive antibody response to EBP2 (aOR = 1.95, 95% CI = 1.51–2.51, P<0.0001) and in 1.2 times for antibodies against DEKnull-2 (DEKnull-2, aOR = 1.24, 95% CI = 1.01–1.40, P = 0.001), confirming the influence of exposure to malaria on the IgG antibody response to these *P*. *vivax* ligands.

## Discussion

Efforts to prioritize *P*. *vivax* antigen-combination for vaccine development suggest an additive effect of antibodies against DBPII and the recently described EBP2 [26,27]. While these findings are promising, a major drawback was the observation that the protective effect of the EBP2- DBPII combination was DBPII allele-specific [27]. Considering that available data on naturally acquired immunity to the EBP2 is still scarce [26,27,34], we investigated here whether a primary *P*. *vivax* exposure induces antibodies against EBP2 that could be boosted by *P*. *vivax* relapses or recurrent infections. Our experimental approach also involved an engineering DBPII immunogen (DEKnull-2) whose immune response has been associated with strain-transcending antibodies [33]. The results showed that a primary *P*. *vivax* infection was not sufficient to induce significant IgM/ IgG antibodies to either EBP2 or DEKnull-2. Unexpectedly, EBP2 antibodies were not boosted by *P*. *vivax* recurrent infections following antimalarial treatment; at the time of the outbreak, it was not possible to differentiate recrudescence due to therapeutic failures or relapses arising from persistent liver stages of the parasite (hypnozoites) [31]. On the other hand, IgG antibodies against DEKnull-2 were boosted by recurrent blood-stage *P*. *vivax* infections, as well as antibodies against the homologous DBPII variant linked to the outbreak. These results with DEKnull-2 are of interest because we and others have demonstrated before that naturally acquired DBPII antibodies tend to be short-lived and biased towards strain-specific responses [31,32]. Of Interest, sustained DEKnull-2 IgG responses had also been described in long-term malaria exposed individuals [41], reinforcing the potential of DEKnull-2 as vaccine target.

Although the reasons for the absence of anti-EBP2 booster in *P*. *vivax* primo infections are not known, it is possible to speculate that polymorphisms in *ebp2* gene could be a factor, as data from Southeast Asian suggested that region II of EBP2 is highly polymorphic [34]. However, polymorphism in the *ebp2* gene does not seem to explain EBP2 low immunogenicity because the *ebp2* allele from the *P*. *vivax* outbreak showed high sequence identify to the reference Cambodian isolated C127 (used here as recombinant protein). Specifically, these alleles differed by a single nucleotide polymorphism (G1057A). A more plausible explanation to the low immunogenicity of EBP2 in *P*. *vivax* primo infected may be related to the host cell specificity as we have previously demonstrated that EBP2 binding properties is much more restricted than observed for DBPII, linking preferentially to Duffy-positive immature bone marrow reticulocytes (CD71^high^) [25]. In addition, *Plasmodium* reticulocytes invasion takes less than one minute [8,42,43], therefore, like DBP and other members of the DBL family, EBP2 may be released “just in time” for reticulocyte attachment and/or junction formation and invasion [16,44,45]. Consequently, multiple *P*. *vivax* infections are required to induce a significant and specific antibody response. This evidence supports our finding that a low EBP2 immunogenicity after a first *P*. *vivax* infection is followed by high recognition (>90%) after long-term malaria exposure in the Amazonian area. These results, although there are limitations of small number of sample size from the *P*. *vivax* outbreak, were reinforced by related studies that showed EBP2 was poorly immunogenic in PNG children but anti- EBP2 antibodies levels were positively correlated with age and cumulative exposure [27]. Considering that EBP2/ DBPII antibody-combinations are associated with reduced risk of clinical disease [26,27], and anti-EBP2 antibodies can block EBP2-erythrocyte binding [25], further characterization of EBP2 including large number of sample size, other populations and functional studies to evaluate the potential for EBP2 IgG antibodies to block *P*. *vivax* reticulocyte invasion are necessary to further validate EBP2 as a potential candidate to partner with DBPII and related antigen, DEKnull-2, in a multivalent blood-stage vaccine against *P*. *vivax*.

## Conclusion

Taken together, our results showed that EBP2 was poorly immunogenic after a single and brief *P*. *vivax* exposure, but it is highly immunogenic after long-term malaria exposure. Finally, the findings further supported the potential of DEKnull-2 as a vaccine candidate, as in non-immune individuals anti-DEKnull-2 IgG antibodies were boosted after brief exposure to *P*. *vivax* blood stages.

## Supporting information

S1 Fig Depiction of primers for sequencing of the DBL domain of EBP2 of *P*. *vivax* Outbreak isolate. To sequence the full Duffy Binding-like (DBL) domain of EBP2, three sets of primers were designed to amplify three overlapping fragments. Positions of primers are indicated (Forward and Reverse): Fragment 1 in pink (position 201bp to 870bp), Fragment 2 in blue (position 712bp to 1262bp) and Fragment 3 in green (position 996bp to 1618bp).
(TIF)

S2 Fig IgM and IgG antibody profile of *P*. *vivax* DBPII after first *P*. *vivax* exposure. (A) Frequency and level of IgM and IgG antibody against *P*. *vivax* DBPII-outbreak in individuals with acute *P*. *vivax* infection (Case, n = 16), and relatives and/ or neighbors without malaria symptoms (Non-Case, n = 22). The IgM and IgG antibody responses were evaluated at the *P*. *vivax* outbreak (BL, baseline), 3, 6 and 12 months after the outbreak for both the Case and Non-Case groups. Serum reactivity was expressed as ELISA Reactivity Index (RI). Percentage (%) of antigen-specific IgM and IgG positive was expressed at the top of the graph. The dashed line represents RI = 1. Samples with RI > 1.0 are considered positive. (B) Heatmap of influence of *P*. *vivax* recurrence on the IgM and IgG antibody responses to *P*. *vivax* DBPII. Individuals who experienced first *P*. *vivax* malaria infection (Cases) were grouped into: (i) Recurrence (n = 6)–individuals who experienced one or two additional recurrent *P*. *vivax* infection; and (ii) No-recurrence (n = 10)–individuals who did not have additional blood-stage *P*. *vivax* infection. The color gradient indicates the intensity of IgM (red) and IgG (blue) antibody levels categorized by tercile in High (Upper tercile), Medium (Second tercile) and Low (First tercile) for each protein. The time points of follow-up study and recurrent *P*. *vivax* infection moment were indicated at the heatmap.
(JPG)

S3 Fig Influence of *P*. *vivax* parasitemia on booster IgG antibody response to both DEKnull-2 and DBPII among individuals experienced *P*. *vivax* recurrence. (A) Parasitemia (parasites/μL) (dashed line) and IgG antibody level against DEKnull-2 (continuous line), expressed by Reactivity index (RI) for each subjects experienced *P*. *vivax* recurrence; (B) Parasitemia (parasites/μL) (dashed line) and IgG antibody level against DBPII (continuous line), expressed by Reactivity index (RI) for each individual experienced *P*. *vivax* recurrence (3 and 6 months after the first *P*. *vivax* infection). The x-axis represents the time of *P*. *vivax* recurrence (3 and 6 months after the first *P*. *vivax* infection).
(TIFF)

S1 Table Risk factors associated with immunological response against *P*. *vivax* blood stage vaccine antigens. (DOCX)

S1 Data Demographic, epidemiological data and immunological response against *P*. *vivax* blood stage vaccine antigens of the study population. (XLS)
