# Supplementary figures and images for: Antibody response to a new member of the DBL family (EBP2) after a brief Plasmodium vivax exposure
Source: PLoS Negl Trop Dis. 2022 Jun 17;16(6):e0010493. doi: 10.1371/journal.pntd.0010493 (PMC9205486; doi:10.1371/journal.pntd.0010493)

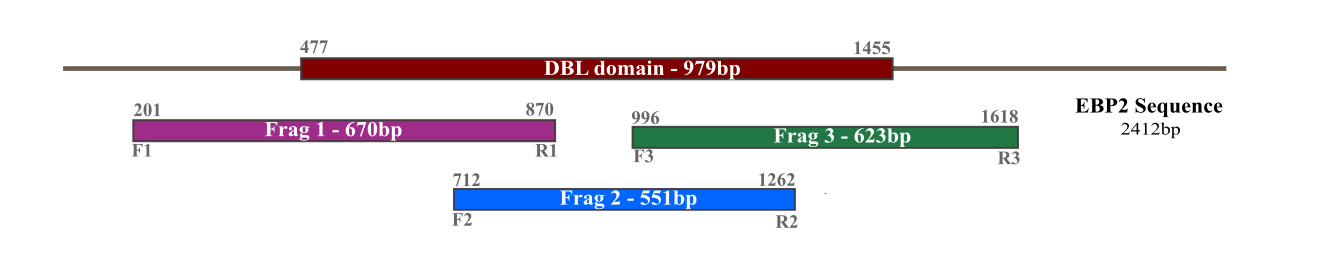

Supplement: S1 Fig — To sequence the full Duffy Binding-like (DBL) domain of EBP2, three sets of primers were designed to amplify three overlapping fragments. Positions of primers are indicated (Forward and Reverse): Fragment 1 in pink (position 201bp to 870bp), Fragment 2 in blue (position 712bp to 1262bp) and Fragment 3 in green (position 996bp to 1618bp). (TIF) [file pntd.0010493.s001.tif]

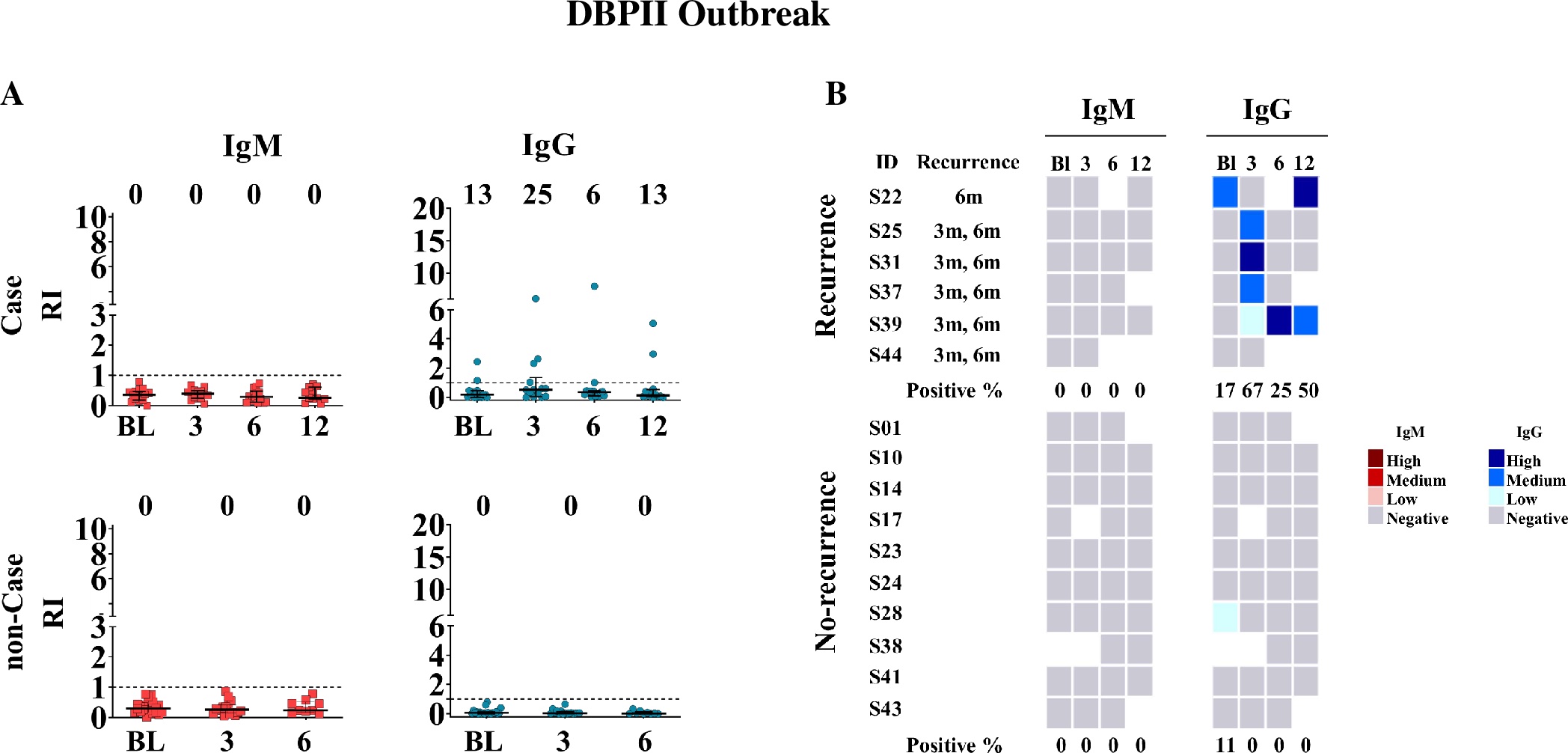

Supplement: S2 Fig — (A) Frequency and level of IgM and IgG antibody against P. vivax DBPII-outbreak in individuals with acute P. vivax infection (Case, n = 16), and relatives and/ or neighbors without malaria symptoms (Non-Case, n = 22). The IgM and IgG antibody responses were evaluated at the P. vivax outbreak (BL, baseline), 3, 6 and 12 months after the outbreak for both the Case and Non-Case groups. Serum reactivity was expressed as ELISA Reactivity Index (RI). Percentage (%) of antigen-specific IgM and IgG positive was expressed at the top of the graph. The dashed line represents RI = 1. Samples with RI > 1.0 are considered positive. (B) Heatmap of influence of P. vivax recurrence on the IgM and IgG antibody responses to P. vivax DBPII. Individuals who experienced first P. vivax malaria infection (Cases) were grouped into: (i) Recurrence (n = 6)–individuals who experienced one or two additional recurrent P. vivax infection; and (ii) No-recurrence (n = 10)–individuals who did not have additional blood-stage P. vivax infection. The color gradient indicates the intensity of IgM (red) and IgG (blue) antibody levels categorized by tercile in High (Upper tercile), Medium (Second tercile) and Low (First tercile) for each protein. The time points of follow-up study and recurrent P. vivax infection moment were indicated at the heatmap. (JPG) [file pntd.0010493.s002.jpg]

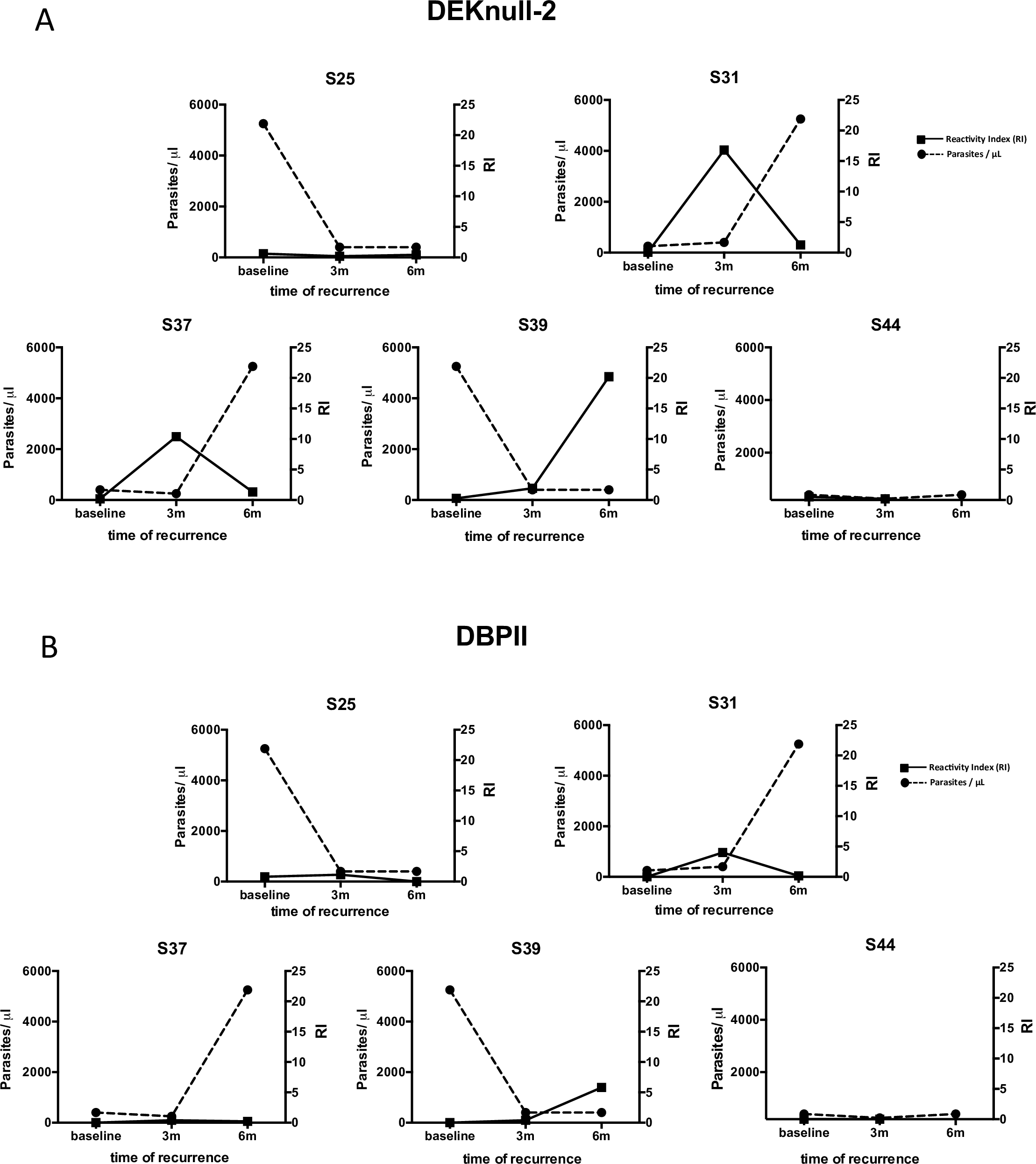

Supplement: S3 Fig — (A) Parasitemia (parasites/μL) (dashed line) and IgG antibody level against DEKnull-2 (continuous line), expressed by Reactivity index (RI) for each subjects experienced P. vivax recurrence; (B) Parasitemia (parasites/μL) (dashed line) and IgG antibody level against DBPII (continuous line), expressed by Reactivity index (RI) for each individual experienced P. vivax recurrence (3 and 6 months after the first P. vivax infection). The x-axis represents the time of P. vivax recurrence (3 and 6 months after the first P. vivax infection). (TIFF) [file pntd.0010493.s003.tiff]
