# Supplementary material for: Antibody response to a new member of the DBL family (EBP2) after a brief Plasmodium vivax exposure
Source: PLoS Negl Trop Dis. 2022 Jun 17;16(6):e0010493. doi: 10.1371/journal.pntd.0010493 (PMC9205486; doi:10.1371/journal.pntd.0010493)
Supplement: S1 Table — (DOCX) [file pntd.0010493.s004.docx]

**S1 Table. Risk factors associated with immunological response**

**against *P. vivax* blood stage vaccine antigens**

| **Variables** | **aOR (95% CI*)** | ***P*-value** |
| --- | --- | --- |
| **EBP2** |  |  |
| *Age* | 1.01 (0.97 - 1.04) | 0.52 |
| *Gender* | 0.71 (0.23 - 2.15) | 0.54 |
| *Previous malaria*** | **1.95 (1.51 - 2.51)** | **<0.0001** |
| *Months since last malaria* | 1.09 (0.98 - 1.21) | 0.09 |
| **DEKnull-2** |  |  |
| *Age* | 1.00 (0.97 - 1.02) | 0.93 |
| *Gender* | 1.30 (0.57 - 2.96) | 0.52 |
| *Previous malaria* | **1.24 (1.01 - 1.40)** | **0.0004** |
| *Months since last malaria* | 0.99 (0.94 - 1.05) | 0.91 |

In bold, there was statistical significance level of 5% (*P*-value <0.05).

*The odds ratio (OR), respective 95% confidence intervals (95% CI).

** Number of previous malaria episodes
